# Environmental Sampling for Avian Influenza Virus A (H5N1) in Live-Bird Markets, Indonesia

**DOI:** 10.3201/eid1612.100402

**Published:** 2010-12

**Authors:** Risa Indriani, Gina Samaan, Anita Gultom, Leo Loth, Sri Indryani, Rma Adjid, Ni Luh Putu Indi Dharmayanti, John Weaver, Elizabeth Mumford, Kamalini Lokuge, Paul M. Kelly, Darminto

**Affiliations:** Author affiliations: Indonesian Research Center for Veterinary Science, Bogor, Indonesia (R. Indriani, R. Adjid, N.L.P.I. Dharmayanti, Darminto);; The Australian National University, Canberra, Australian Capital Territory, Australia (G. Samaan, K. Lokuge, P.M. Kelly);; World Health Organization, Jakarta, Indonesia (G. Samaan);; Ministry of Health, Jakarta (A. Gultom, S. Indryani);; Food and Agriculture Organization, Dhaka, Bangladesh (L. Loth);; Food and Agriculture Organization, Hanoi, Vietnam (J. Weaver);; World Health Organization, Geneva, Switzerland (E. Mumford)

**Keywords:** Avian influenza, influenza, H5N1, live bird markets, transmission, environment, viruses, Indonesia, surveillance, research

## Abstract

To identify environmental sites commonly contaminated by avian influenza virus A (H5N1) in live-bird markets in Indonesia, we investigated 83 markets in 3 provinces in Indonesia. At each market, samples were collected from up to 27 poultry-related sites to assess the extent of contamination. Samples were tested by using real-time reverse transcription–PCR and virus isolation. A questionnaire was used to ascertain types of birds in the market, general infrastructure, and work practices. Thirty-nine (47%) markets showed contamination with avian influenza virus in >1 of the sites sampled. Risk factors were slaughtering birds in the market and being located in West Java province. Protective factors included daily removal of waste and zoning that segregated poultry-related work flow areas. These results can aid in the design of evidence-based programs concerning environmental sanitation, food safety, and surveillance to reduce the risk for avian influenza virus A (H5N1) transmission in live-bird markets.

## Abstract 2

TOC Summary: This method is time and labor efficient and minimizes potential risk for virus aerosolization.

Food markets that offer both poultry meat and live birds either for sale or for slaughter are collectively referred to as live-bird markets (LBMs). LBMs are part of the supply chain and are essential for maintaining the health and nutritional status of rural and urban populations, especially in developing countries (*1**,**2*). However, LBMs provide optimal conditions for the zoonotic transfer and evolution of infectious disease pathogens because they provide major contact points between humans and live animals (*3**,**4*).

Studies in Hong Kong Special Administrative Region, People’s Republic of China; other areas of China; Indonesia; and the United States have shown that LBMs can harbor avian influenza viruses (AIVs), including highly pathogenic influenza virus A (H5N1), and have been associated with human infection (*4**–**9*). Continual movement of birds into, through, and out of markets provides opportunity for the introduction, entrenchment, and dissemination of AIVs. Most studies have focused on testing live birds rather than environmental sites in the LBMs (*6**,**7**,**10*). However, a study in New York, NY, that tested environmental sites for AIV (H7N2) found that virus could be isolated from samples from floors, walls, and drains from the poultry areas of LBMs (*8*). The study also found that despite the ongoing influx of infected birds into LBMs, the level of environmental contamination decreased with routine cleaning and disinfection. Another study in Hong Kong LBMs showed that AIV (H9N2) could be isolated at higher rates from poultry drinking water than from samples of bird fecal droppings (*11*). Environmental aspects of LBMs are needed for an avian influenza control program for 2 reasons. First, a contaminated environment can provide a continuing source of virus transmission, in which healthy birds coming into the market may become infected and persons working in or visiting the market may also be exposed. Second, ongoing surveillance programs in LBMs based on environmental sampling are more likely than those based on invasive bird testing to be acceptable to traders and stall vendors. Environmental sampling is also safer for public health officers and veterinary health officers than handling and sampling live birds that may be infected with AIV.

In this study, we aimed to identify the environmental sites commonly contaminated by AIV (H5N1) in LBMs in Indonesia. Identifying these sites is the first step in the design of evidence-based environmental sanitation, food safety, and surveillance programs to reduce the risk for virus transmission and to develop environmental surveillance programs to monitor LBM contamination status.

## Methods

Three provinces in the western part of Java Island in Indonesia participated in the study: Jakarta, Banten, and West Java (Figure). Eighteen districts in these provinces were selected on the basis of their proximity to the laboratory, high levels of avian influenza activity in farmed birds (Ministry of Agriculture, unpub. data), and high number of LBMs available for study (n = 300). The required sample size was 73 markets based on an estimated disease prevalence of 50% and a maximum error of 10% at 95% confidence. We based our assumption that 50% of LBMs would be contaminated with AIV (H5N1) on results from a previous study in US LBMs in 2001 (*12*). This study found that 60% of markets tested positive for AIV (H7N2) virus in areas in which the virus was endemic. To account for nonresponse, we increased the total sample size to 83 LBMs. We selected markets for inclusion in the study using systematic sampling. On the basis of a sampling frame of 300 markets, every fourth market (the sampling interval) was selected from a list of all the markets. A random numbers table was used to determine the starting point for selection of the 83 markets from the list. Diagnostic specimens and data were collected during October 2007–March 2008. These months have high rainfall and high AIV transmission according to data gathered during 2005–2007 about AIV (H5N1) outbreaks in farmed birds (Ministry of Agriculture, unpub. data).

**Figure:**
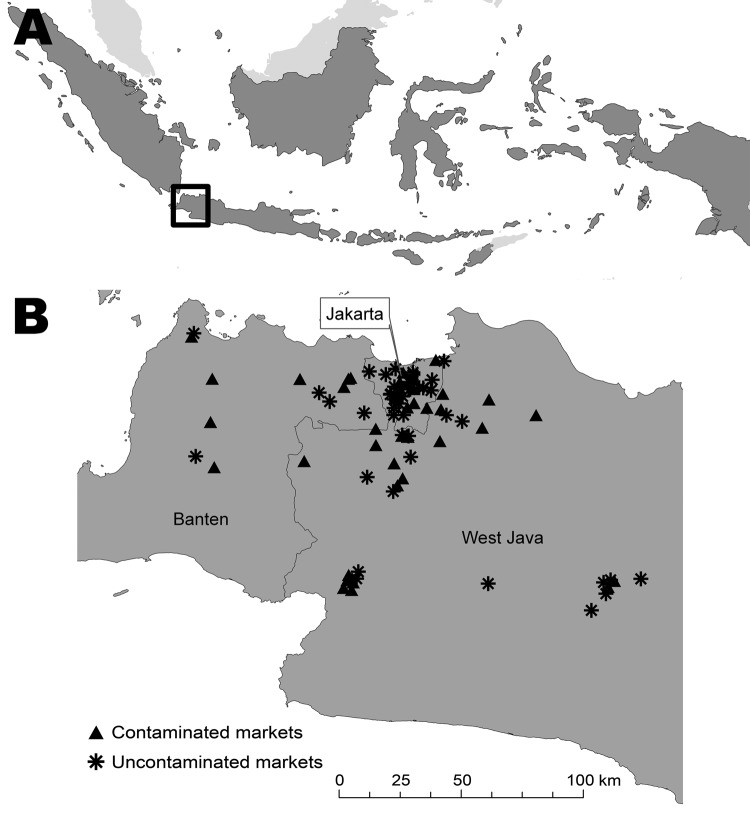
A) Area of study of avian influenza virus A (H5N1) contamination in live-bird markets (black box), western Java, Indonesia, 2007–2008. B) Distribution of contaminated and uncontaminated markets in the study area.

A structured questionnaire containing 42 questions to assess risk factors for AIV (H5N1) contamination was developed. Responses to questions were obtained through visual inspection of each LBM and through an interview with the manager of the participating LBM. The questions sought information about volume of poultry in the LBM and the infrastructure in the delivery, holding, slaughter, sale, and waste-disposal zones of the market. These 5 zones reflect general demarcation of work flow and activities relating to poultry in LBMs (*13*). Questions about the sanitation and slaughtering practices were also included.

Questionnaire validation was conducted by members of a study advisory team. The team comprised 2 food safety/environmental health officers from the Ministry of Health, a communicable disease epidemiologist from the World Health Organization, a veterinary epidemiologist from the Food and Agriculture Organization, and 2 virologists from the Ministry of Agriculture in Indonesia. The questionnaire was tested in 3 LBMs in West Java province to ensure coherence, appropriate use of terminology, and high face validity. The same markets were also inspected to ensure that the questionnaire addressed all aspects of the poultry-related work flow in the 5 poultry zones and relevant infrastructure. Members of the study advisory team trained 3 study data collection teams in questionnaire administration and sample collection procedures.

To select the environmental sites to be sampled in each LBM, the study advisory team visually inspected 3 markets and reviewed the literature to identify LBM sites commonly contaminated with AIVs or similar pathogens. Sites sampled in previous studies for AIV included floors, drains, and water troughs (*8**,**11**,**12*). In this study, 27 sites were selected for environmental sampling (Table 1). The sites represented different poultry-related work activities: 3 sites related to delivery of birds into LBMs, 7 in the bird-holding zone, 9 in the slaughter zone, 6 in the sale zone, and 2 in the waste-disposal zone. Because of variation in LBM infrastructure and processes, each LBM did not necessarily have all 27 sites. Samples were collected from as many of the 27 sites as were available in each LBM.

**Table 1:** Environmental sites in LBMs contaminated by influenza virus A (H5N1) as detected by RT-PCR and virus isolation, Indonesia, 2007–2008*

Poultry zone	Site no.	Environmental site	RT-PCR–positive/markets tested (%), N = 1,862	VI–positive/RT-PCR positive, n = 280	LBMs positive for zone
Delivery	1	Inside cages on truck	6/45 (13.3)	1/6	11
	2	Floor in delivery area	6/49 (12.2)	0/6	
	3	Water run-off in delivery area	4/38 (10.5)	0/4	
Holding	4	Poultry cage floors	6/79 (7.6)	0/6	24
	5	Holding area floor	8/80 (10)	1/8	
	6	Water run-off	11/72 (15.3)	0/11	
	7	Poultry feeding bottle water	8/67 (11.9)	0/8	
	8	Poultry feeding basket food	6/72 (8.3)	0/6	
	9	Handles to poultry cages	9/79 (11.4)	0/9	
	10	Inside of waste bins	10/59 (16.9)	0/10	
Slaughter	11	Handles of knives used for slaughtering	8/75 (10.7)	1/8	29
	12	Basket holding dying chickens	8/71 (11.3)	2/8	
	13	Floor in slaughter area	10/77 (13)	0/10	
	14	Chopping or slaughtering board	14/71 (19.7)	2/14	
	15	Processing table after de-feathering	15/70 (21.4)	0/15	
	16	Baskets holding poultry meat	14/70 (20)	1/14	
	17	Drain path	12/75 (16)	0/12	
	18	Tap handles in slaughter area	7/65 (10.8)	0/7	
	19	Waste bin	13/71 (18.3)	1/13	
Sale	20	Chopping boards	15/80 (18.8)	1/15	30
	21	Scales	12/57 (21.1)	0/12	
	22	Knife handles	12/78 (15.4)	1/12	
	23	Waste bins	10/60 (16.7)	1/10	
	24	Wet cloths for cleaning surfaces	14/78 (17.9)	0/14	
	25	Tables for poultry display	19/80 (23.8)	0/19	
Waste disposal	26	Area waste-disposal bin	15/78 (19.2)	1/15	9
	27	Wet cleaning mops	8/66 (12.1)	0/8	
Total positive			280 (15)	13 (4.6)	

*LBM, live-bird market; RT-PCR, reverse transcription–PCR; VI, virus isolation.

For each of the 27 sites, 6 swab specimens were collected and pooled. Each pool (vial) consisted of a maximum of 3 swabs. The data collection teams were instructed to increase the representativeness of the samples by swabbing different locations for each environmental site. For example, if the market had 6 poultry stalls, each with its own scale for weighing poultry, then teams collected 1 swab from each scale and pooled them into 2 pools of 3 swabs each. Swab specimens were pooled in the market, and swabs remained inside the vials until testing. The data collection teams were instructed to focus on visibly dirty, moist, or difficult-to-clean surfaces in an effort to increase the sensitivity of the sampling.

Sample collection, pooling, transportation, and storage were based on techniques used in previous studies (*10**,**12*). Each data collection team comprised 3 persons, 2 of whom collected samples and 1 administered the questionnaire. To reduce the risk for cross-contamination during sample collection, teams changed disposable gloves and shoe covers between each of the 5 LBM poultry zones. Sterile cotton-tipped swabs were used to collect all samples, and samples were placed in viral transport media and transported immediately back to the laboratory on frozen gel packs. The viral transport media consisted of Dulbecco modified Eagle medium (Sigma-Aldrich, St. Louis, MO, USA) with 1,000 IU penicillin and gentamicin, and 1% fetal buffer serum (*14*). Samples were stored in the laboratory at –70°C until tested.

RNA extraction, cDNA synthesis, and real-time reverse transcription–PCR (RT-PCR) were used as described (*15*). Virus isolation methods have also been described (*16*) but in general involved supernatants from a 1,000-µL sample homogenized by vortex and centrifuged at 2,500–3,000 rpm into 9- to 10-day-old specific pathogen–free eggs. Those positive in the hemagglutination assay were tested by hemagglutination-inhibition test with reference antiserum (A/chicken/West Java/Hamd/2006).

The degree of association between AIV (H5N1) positivity in the 5 LBM poultry zones was determined by using Spearman rank correlation. To assess risk factors for environmental virus (H5N1) contamination, we estimated odds ratios (ORs) using multivariable logistic regression analyses, where variables with p<0.1 from the univariate analyses were included in the initial model. A backward stepwise variable–selection strategy was used to construct a final model with a significance level of p<0.05. The Hosmer and Lemeshow test and the residual χ^2^ goodness-of-fit test were used to assess model stability. Microsoft Excel (Microsoft, Redmond, WA, USA), Epi Info (Centers for Disease Control and Prevention, Atlanta, GA, USA), and Stata version 10.0 (StataCorp, College Station, TX, USA) were used for the descriptive and statistical analyses.

Approval for the study was obtained from the Health Research Ethics Committee at the Indonesian Ministry of Health and the Australian National University Human Research Ethics Committee. Permission was obtained from LBM managers before participation in the study.

## Results

### LBM Demographics and Practices

All 83 LBMs selected participated in the study; 62 (75%) were located in urban and 21 in rural areas. LBMs were from 16 districts in 3 provinces: 31 (38%) from Jakarta province, 11 (13%) from Banten province, and 41 (49%) from West Java province (Figure). Most (49 [59%]) LBMs were retail markets, 10 (12%) were wholesale only, and 24 (29%) were a combination of retail and wholesale. Most (82 [99%]) LBMs operated daily, with the same vendors operating in the same stalls.

Most LBMs received their poultry from commercial farms (71 [86%]), and some also sourced poultry from small-scale holders (36 [43%]). Most (42 [51%]) LBMs had medium-sized poultry areas (11–50 poultry cages), and 21 (25%) had large poultry areas (>50 cages). LBMs had village free-ranging chickens (69 [83%]), fighting cocks (13 [16%]), broilers (67 [81%]), spent hens (24 [29%]), Muscovy ducks (48 [58%]), ducks other than Muscovy (32 [39%]), and pigeons (16 [19%]). Most (71 [86%]) LBMs generally kept live poultry in the market for a few days until sold, housing them overnight in cages.

Forty-eight (58%) LBMs reported monthly or more frequent visits from animal/human health personnel to inspect the poultry zones. Eight (10%) LBMs reported that live birds were tested periodically (less frequently than weekly) for AIV infection. For cleaning and sanitation, 80 (96%) LBMs reported washing poultry zones daily, and 55 (66%) applied detergent or disinfectant daily.

### Laboratory Findings

Thirty-nine (47%) LBMs had evidence of contamination. For 17 (44%) of these, <5 environmental sites were positive for AIV (H5N1) by real-time RT-PCR. For each of 22 (56%) LBMs, >6 environmental sites were positive.

The environmental sites most heavily contaminated were in the slaughter and sale zones (Table 1). In the slaughter zone, the most contaminated sites were the poultry-processing tables (21%), baskets holding poultry meat (20%), and chopping boards (20%). In the sale zone, the most contaminated sites were the tables for carcass display (24%) and scales (21%). Another commonly contaminated site was the waste-disposal bin in the waste-disposal zone (19%). In most cases, this bin is not an enclosed bin but rather was a dedicated uncovered floor space where remnants are dumped daily and collected weekly by the local government rubbish collection team.

Thirteen viruses were isolated from LBMs, most frequently from the slaughter zone (7 of 13 viruses isolated, Table 1). All isolated viruses came from 6 LBMs, from which 1–4 viruses were isolated per LBM.

From the zones contaminated in each LBM (Table 1), we calculated correlations between different zones. Contamination in preceding LBM poultry zones correlated with contamination in the subsequent zones (Table 2). Correlations were high between holding and slaughter zones, slaughter and sale zones, and sale and waste-disposal zones.

**Table 2:** Correlation coefficient of influenza virus A (H5N1) positivity between 5 poultry zones in live-bird markets, Indonesia, 2007–2008

Site	Delivery	Holding	Slaughter	Sale	Waste disposal
Delivery	1				
Holding	0.84	1			
Slaughter	0.82	0.89	1		
Sale	0.63	0.84	0.87	1	
Waste disposal	0.50	0.26	0.52	1	1

### Risk Factors for Contamination

We assessed risk factors for AIV (H5N1) contamination in LBMs. We compared exposures in 39 LBMs with a minimum of 1 contaminated environmental site to 44 LBMs with no contamination. From the univariate analyses, several exposures predicted AIV (H5N1) contamination in LBMs (Table 3). LBMs with wooden tables, Muscovy ducks, or >200 ducks other than Muscovy were at greater risk for AIV (H5N1) contamination, as were LBMs in West Java province.

**Table 3:** Comparison of exposures in LBMs with AIV (H5N1) environmental contamination and in LBMs with no environmental AI (H5N1) contamination, Indonesia, 2007–2008*

Exposure	No. positive markets, n = 39	No. negative markets, n = 44	OR (95% CI)	p value
No. ducks other than Muscovy in LBM				
<11	8	11	Reference group	
11–100	12	16	1.03 (0.32–3.35)	0.959
101–200	2	2	4.13 (0.16–11.95)	0.773
>200	10	2	6.88 (1.17–40.38)	0.033
Muscovy ducks	28	20	3.05 (1.22–7.63)	0.017
Pigeons	11	5	3.06 (0.96–9.81)	0.059
Clear zoning in LBM	3	10	0.28 (0.07–1.11)	0.072
Wooden tables	23	12	3.83 (1.53–9.62)	0.004
Slaughtering in LBM	36	34	3.53 (0.89–13.93)	0.072
Daily solid waste disposal	24	35	0.41 (0.16–1.09)	0.075
Mixing of species in same cage	13	6	2.92 (0.98–8.70)	0.055
Cages stacked vertically	25	33	0.38 (0.13–1.10)	0.069
Province				
Jakarta	23	8	Reference group	
West Java	25	16	4.49 (1.62–12.46)	0.004
Banten	6	5	3.45 (0.82–14.47)	0.090
Multivariable analysis†				
Clear zoning in LBM			0.16 (0.03–0.86)‡	0.030
Slaughtering in LBM			6.43 (1.01–40.82)‡	0.048
Daily solid waste disposal			0.20 (0.06–0.69)‡	0.010
Province				
Jakarta			Reference group	
West Java			6.83 (2.01–23.19)‡	0.002
Banten			2.94 (0.59–14.69)‡	0.190

*LBM, live-bird market; AI, avian influenza; OR, odds ratio; CI, confidence interval. †Final model with 4 variables, no. observations = 83, goodness-of-fit tests: residual χ^2^, p = 0.38; Hosmer and Lemeshow test, p = 0.45. ‡Adjusted OR.

Six other exposures approached significance, either as protective factors or as risk factors. LBMs that disposed and removed solid waste daily (OR 0.41, 95% confidence interval [CI] 0.16–1.09); had zoning that clearly segregated poultry delivery, holding, slaughter, sale, and waste-disposal areas (OR 0.28, 95% CI 0.07–1.11); or stacked poultry cages vertically rather than side by side (OR 0.38, 95% CI 0.13–1.10) had less risk for avian influenza virus (H5N1) contamination. LBMs with pigeons (OR 3.06, 95% CI 0.96–9.81), mixed bird species in the same cages (OR 2.92, 95% CI 0.98–8.70), or slaughtered birds in the market (OR 3.53, 95% CI 0.89–13.93) were more likely to be contaminated.

None of the 9 other variables considered in the study were associated with AIV (H5N1) contamination in LBMs (data not shown). These included the LBM trading category (wholesale, retail, or combination), days operational per week, chicken population in LBM, source of chickens (small-scale backyard farmers, commercial farms, or combination), inspection from authorities, use of detergent during cleaning, mixing poultry arriving on different days in the same cages, average length of poultry stay in LBM, and whether poultry were removed from stalls before cleaning.

From the univariate analyses, 10 variables were significant at p<0.1. However, the ducks other than Muscovy variable was removed from the multivariate analyses because of its collinearity with another variable (presence of Muscovy ducks, r>0.4). Nine variables were considered for the multivariate analyses. The final multivariable logistic regression model had 4 variables, of which 2 were independent risk factors for subtype H5N1 contamination in LBMs (Table 3). They were location in West Java province (adjusted OR [aOR] 6.83, 95% CI 2.01–23.19) and bird slaughtering in the LBM (aOR 6.43, 95% CI 1.01–40.82). Two variables were independent protective factors: zoning of poultry activities in LBMs (aOR 0.16, 95% CI 0.03–0.86) and daily disposal of solid waste (aOR 0.2, CI 95% 0.06–0.69).

## Discussion

We have demonstrated extensive environmental contamination in LBMs with the AIV (H5N1) in Indonesia. Nearly 50% of LBMs in AIV (H5N1)–endemic districts were positive, with all 5 poultry zones affected. The study identified environmental points of contamination and protective and risk factors for contamination. This study provides baseline information for 2 aspects that can aid in control of AIV (H5N1) in LBMs: 1) development of routine monitoring and surveillance programs and 2) structural interventions and work flow modifications to minimize risk for contamination.

Our findings provide further evidence that environmental contamination with AIVs is not uncommon (*8**,**14*). Poultry water, drains, tabletops, cages, tablecloths, utensils, bins, and floors were all contaminated. Environmental sites most commonly contaminated were located in slaughter zones and zones where carcasses were taken after slaughtering, such as the sale and waste-disposal zones. This contamination can be expected because slaughtering generates droplets that may contain viral particles and exposes internal organs with potentially high viral loads. Even if slaughtering is conducted in a separate zone, contamination can spread to the sale and waste-disposal zone through the carcasses and through the process of evisceration usually conducted in both slaughter and sale stalls.

We found rates of contamination in water from poultry feeding bottles similar to those from the study in Hong Kong on AIV (H9N2) (11% and 7% markets with contamination respectively, p = 0.12) (*11*). Even though AIVs were detected from poultry drinking water, our study suggests that other environmental sites are more efficient for monitoring AIV (H5N1) in markets. Processing tables and baskets holding freshly cut poultry meat in the slaughter area, as well as display tables and scales in the sale area, were positive in 20 (24%) LBMs surveyed.

The risk and protective factors we identified complement findings from previous studies. Daily disposal and removal of waste from the market is part of routine environmental cleaning and sanitation and eliminates AIV reservoirs (*8*). Segregating poultry-related activities into zones limits virus spread (*17*). Vertical stacking of cages can limit transmission because trays between layers of birds prevent the scatter of fecal matter. These results add evidence to the World Health Organization current recommendation that waste trays should be used to segregate stacked cages in markets to prevent cross-contamination (*13*).

LBMs in West Java province had a higher risk for contamination than did other provinces. This risk probably is due to greater AIV (H5N1) disease activity in the province. Surveillance activities during 2006–2008 showed that West Java had a 4.7% outbreak detection rate compared with rates in Banten (4%) and Jakarta (0.2%) (*18*). Furthermore, in West Java province chicken density is high: 14,000 birds/km^2^ compared with densities in the neighboring provinces Banten and Jakarta (3,900 birds/km^2^ and 400 birds/km^2^, respectively) (*19*). Poultry density data are commonly used as a proxy for disease activity where areas of high poultry density have the highest risk for an outbreak (*20**,**21*).

Several issues need to be considered regarding our finding of low virus isolation rates compared with real-time RT-PCR–positive rates. Virus isolation detects viable virus, whereas real-time RT-PCR detects small stretches of nucleic acid, even if the larger genomic RNA is inactivated. This makes real-time RT-PCR a more sensitive detection tool but does not provide information about virus viability. Samples obtained from the environment may be less suitable than animal samples for virus isolation techniques. Organic matter, duration and temperature of exposure, and humidity can all affect virus survival outside the animal host (*22*). Three studies conducted in LBMs tested environmental samples and bird samples by using virus isolation (*8**,**10**,**23*). Only 1 of these studies stratified the avian influenza detection rates by type of sample (bird vs. environment) (*8*); that study found that from 12 LBMs, 11 were positive for avian influenza in bird samples compared with only 5 positive in environmental samples. These results were based on a small sample of LBMs, and real-time RT-PCR was not conducted. Therefore, to determine the suitability of virus isolation for environmental samples, we recommend that future studies compare real-time RT-PCR–positive rates to virus isolation rates in both environmental swab and bird samples.

Risk and protective factors identified in this study, together with findings from other studies, can assist in developing environmental or behavioral interventions to reduce AIV transmission in LBMs. Previous studies have shown that regular cleaning with detergents, including free chlorine concentrations typically used in drinking water treatment, can rapidly decontaminate surfaces from AIVs (*8**,**24*). Previous studies also have shown that periodic market rest days coupled with thorough cleaning can minimize the reservoir of AIV in LBMs (*4**,**12**,**25*). These messages have been disseminated to LBMs throughout Indonesia and formed the basis of the Ministry of Health Decree in 2008 on building healthy food markets (*26*).

For a more systematic food safety monitoring system, this study will be used to develop a risk-based approach for AIV risk reduction in LBMs in Indonesia (*27*). The contamination sites and risk factors will be used to determine critical control points and critical limits for intervention. LBM operators, stall vendors, and other stakeholders (e.g., sanitarians and public health officers) will need to be provided with simple monitoring plans to reduce the risk for contamination. Such monitoring plans are expected to have an impact not only on AIV (H5N1) but also on other viruses and bacteria commonly associated with food safety for poultry products.

In addition to tools for disease control, the study findings can aid AIV (H5N1) surveillance activities in LBMs. Commonly contaminated environmental sites in LBMs can form the basis of an environmental sampling strategy for detection of AIV (H5N1) in LBMs. Environmental sampling is more beneficial than live-bird sampling because it is less time and labor intensive and eliminates the need to handle and restrain live birds. Environmental sampling reduces the potential for virus aerosolization and the risk for infection for persons collecting the samples or standing nearby. Further work is needed to assess the adequacy of environmental sampling for surveillance in LBMs under different conditions, especially because detection sensitivity will vary by AIV (H5N1) prevalence in farms supplying the birds.

A limitation of this study is that the observation of environmental contamination was based on a cross-sectional survey in which LBMs were sampled only once. We recommend that future studies observe persistence of the virus over time in the various environmental sites. Reports from market managers and vendors about inspection and cleaning practices in the LBMs were not verified during the course of the study. These activities may have been overreported because respondents may have wanted to report what they perceived interviewers wanted to hear. Because of the high cost associated with the field and laboratory work for such studies, studies should focus on a small number of markets and collect in-depth information about contamination trends and associated risk factors, as well as data on other indicator organisms, such as *Escherichia coli* or *Enterobacteriaceae*, that provide information about general market hygiene. Future work also should evaluate the effects of interventions in markets especially in low-resource settings because this would be of most benefit to low-income and middle-income countries.
